# Neural oscillatory activity during guided and self-generated motor imagery: a comparative case study of a six-time World Cup skateboarder champion and an experienced recreational surfer

**DOI:** 10.3389/fnhum.2026.1964730

**Published:** 2026-10-07

**Authors:** Luciane Aparecida Moscaleski, Alexandre Moreira

**Affiliations:** NeuroSports Lab, Department of Sport, School of Physical Education and Sport, University of São Paulo, São Paulo, Brazil

**Keywords:** electroencephalography (EEG), motor expertise, motor imagery, neural oscillations, sensorimotor representations

## Abstract

This study aimed to examine and compare neural oscillatory activity during guided and self-generated motor imagery in two individuals with contrasting levels of competitive expertise: a six-time World Cup skateboarder champion and an experienced recreational surfer. Following baseline recording, continuous electroencephalographic (EEG) activity (Brain Products GmbH, Gilching, Germany) was acquired during three motor imagery conditions: verbally guided training imagery, verbally guided competition imagery, and self-generated competition imagery. Analyses of frontal alpha asymmetry (FAA), frontal beta asymmetry (FBA), and frontal midline theta (fmθ) showed distinct patterns of frontal oscillatory activity across imagery conditions and between the two athletes. FAA and FBA values were consistently lower in the skateboarder than in the surfer during the imagery conditions, although between-case differences were already evident at baseline. In contrast, fmθ showed a condition-dependent pattern, with higher values in the skateboarder during verbally guided training imagery but lower values during verbally guided and self-generated competition imagery. These case-level observations suggest that frontal oscillatory activity during motor imagery may vary according to imagery condition and individual characteristics. However, because the two participants differed not only in competitive expertise but also in sport discipline, prior imagery training, and trait imagery ability, the observed EEG differences cannot be attributed specifically to motor expertise. Accordingly, these findings should be considered preliminary and hypothesis-generating, providing a basis for future studies examining the relationships among motor expertise, imagery modality, and neural oscillatory activity in larger and more homogeneous samples.

## 1 Introduction

Neuroscience has become a fundamental discipline for understanding the complex interactions underlying brain–brain and brain–body dynamics that govern human movement and athletic performance. The human brain continuously responds to internal and external demands by reorganizing neural circuits across multiple temporal scales, from milliseconds to years, allowing adaptive modifications that support motor learning, decision-making, and skilled performance (Kays et al., 2012; Wolpert et al., 1998).

Consequently, sports performance should no longer be viewed exclusively as the product of muscular or cardiovascular adaptations but rather as the behavioral expression of highly integrated neurophysiological processes. For example, decision-making during sport requires the coordinated activity of multiple brain regions responsible for evaluating environmental information, predicting future events, selecting appropriate motor responses, and continuously updating action plans (Cisek and Kalaska, 2010). The resulting motor behavior therefore reflects the dynamic organization of neural activity rather than isolated muscular execution (Yarrow et al., 2009; Yang and Scholz, 2005; Nakata et al., 2010).

Therefore, interventions specifically designed to influence neural function should not be considered alternatives to conventional training but complementary strategies capable of strengthening the neurocognitive foundations upon which elite performance depends (Fomin and Collins, 2022). Among the available neurocognitive interventions, motor imagery has received considerable scientific attention. Motor imagery can be defined as the internal simulation of an action without overt movement and in the absence of the corresponding sensory input (Morris et al., 2005; White and Hardy, 1998). Rather than representing a simple visual rehearsal, imagery constitutes a multisensory cognitive process involving visual, kinesthetic, proprioceptive, auditory, tactile, and emotional components that recreate the essential characteristics of real performance. Consequently, motor imagery enables athletes to repeatedly activate neural representations associated with skilled movement while avoiding physical fatigue or mechanical stress.

The dynamic brain: the effectiveness of motor imagery is largely supported by the principle of functional equivalence, which proposes that imagined and executed actions recruit substantially overlapping neural substrates. Neuroimaging studies consistently demonstrate that motor imagery activates distributed cortical and subcortical networks, including the supplementary motor area, premotor cortex, dorsolateral prefrontal cortex, posterior parietal cortex, basal ganglia, cerebellum, and sensorimotor regions, that closely resemble those engaged during actual movement execution (Jeannerod, 1994, 2001; Guillot et al., 2012; Hardwick et al., 2018). Although corticospinal output remains below the threshold necessary to generate overt movement, the neural organization underlying imagery closely parallels that observed during physical practice, providing a neurophysiological basis for its widespread application in motor learning, rehabilitation, and high-performance sport.

Accordingly, motor imagery has become an important component of psychological skills training, contributing to technical refinement, tactical preparation, confidence enhancement, emotional regulation, rehabilitation after injury, and optimization of competitive performance (Cumming and Williams, 2012; Munroe-Chandler and Guerrero, 2017). These benefits are particularly relevant in elite sport, where improvements in performance often depend on optimizing neural processing rather than increasing physical capacity alone. In this sense, electroencephalographic studies have consistently shown that elite athletes exhibit oscillatory patterns indicative of enhanced neural efficiency, reflecting more efficient attentional allocation, reduced unnecessary cortical activation, and greater automation of motor control (Del Percio et al., 2011; Del Percio et al., 2009a; Hatfield and Kerick, 2007).

Electroencephalography (EEG) represents one of the most valuable techniques for investigating these processes because of its excellent temporal resolution and sensitivity to oscillatory brain dynamics associated with perception, attention, motor planning, and cognitive workload. Changes in alpha-band oscillations, particularly event-related synchronization and desynchronization, have frequently been associated with attentional regulation, cortical inhibition, motor preparation, and neural efficiency, whereas theta and beta oscillations provide complementary information regarding executive processing, cognitive control, and sensorimotor integration (Cheron et al., 2016). Consequently, EEG offers a unique opportunity to investigate the neurophysiological mechanisms underlying motor imagery in ecologically relevant sport settings.

Importantly, not all imagery procedures engage identical cognitive processes. Different modes of imagery generation may recruit distinct neural mechanisms depending on how the mental representation is constructed. Guided imagery provides standardized external cues that facilitate attentional focus, structured cognitive processing, and controlled simulation of movement. In contrast, self-generated imagery relies predominantly on internally constructed motor representations derived from previous personal experiences and long-term motor memory (Budnik-Przybylska et al., 2021). Because expert athletes possess richer and more refined internal representations of movement, self-generated imagery may preferentially engage neural processes associated with motor automaticity and efficient information processing, whereas guided imagery may require greater executive involvement and attentional control. Despite the growing literature on motor imagery, relatively little is known about how these distinct instructional modalities influence cortical oscillatory dynamics across individuals presenting markedly different levels of sporting expertise.

The present study addresses aimed to compare cortical oscillatory activity during guided and self-generated motor imagery in two individuals representing contrasting levels of expertise: a six-time World Cup skateboarder champion and an experienced recreational surfer. By examining neural oscillations during ecologically valid sport-specific imagery, this study seeks to identify neurophysiological signatures associated with elite motor expertise, functional equivalence, and neural efficiency, while providing further insight into how different imagery modalities modulate cortical dynamics in highly skilled and recreational performers.

## 2 Methods

### 2.1 Participants

This case study included two male participants with markedly different levels of sporting expertise. The first participant was a 51-year-old elite skateboard athlete, a six-time World Cup champion, three-time X Games gold medalist, and three-time European champion. In addition to his extensive competitive career, he had substantial experience with psychological skills training, including mental imagery practice. His imagery ability was confirmed through standardized imagery questionnaires, the results of which are described in the *results* section. The second participant was a 58-year-old experienced recreational surfer who had practiced surfing continuously since the age of 14 years. Although he had no professional competitive background, his more than four decades of surfing experience provided a suitable comparison for examining imagery-related neurophysiological responses in an individual with extensive sport-specific experience but without elite-level competitive expertise. The study was conducted in accordance with the ethical principles of the Declaration of Helsinki. The participants provided written informed consent prior to participation.

### 2.2 Experimental procedure

All experimental sessions were conducted individually by the first author, who has extensive experience in the development and application of mental imagery protocols in sport. Before EEG acquisition, participants completed the 30-item Sport Imagery Questionnaire (SIQ) based on Paivio’s functional model of imagery. The questionnaire comprises five six-item subscales assessing Cognitive Specific, Cognitive General, Motivational Specific, Motivational General-Arousal, and Motivational General-Mastery imagery. Responses are recorded on a 7-point Likert scale, with higher scores indicating more frequent imagery use. The theoretical rationale for these imagery functions follows Paivio’s framework and its subsequent synthesis by Cumming and Williams (2012). This assessment was performed once at the beginning of the experimental session. Following the baseline recording, continuous EEG activity was acquired throughout three mental imagery conditions designed to examine different modes of imagery generation. In the verbally guided conditions, the scenarios were delivered live by the first author using standardized instructions, wording, pacing, and tone of voice to ensure consistency across participants. During all imagery tasks, participants were instructed to keep their eyes closed, remain physically relaxed, minimize body movements, and generate the scenarios as vividly as possible from a first-person (internal) perspective. During the verbally guided competition imagery condition, participants were instructed to imagine themselves immersed in a personally meaningful competition, recreating the visual, kinesthetic, emotional, and contextual aspects of the performance as vividly as possible while maintaining an internal perspective.

#### 2.2.1 The experimental protocol consisted of four consecutive conditions

*Baseline:* resting-state recording with eyes closed for 5 min; *Verbally guided competition imagery*: participants were verbally guided through a standardized competition scenario and instructed to mentally simulate the experience as vividly as possible; *Self-generated competition imagery*: participants independently generated and maintained a vivid mental representation of a personally meaningful competitive performance without external guidance; *Verbally guided training imagery*: participants were verbally guided through a standardized training scenario, including strength and conditioning activities followed by a period of relaxation at home, and were instructed to mentally recreate the complete experience. Each condition lasted 5 min and was separated by a standardized 2-min washout interval to minimize potential carry-over effects between imagery tasks. The conditions were administered in a fixed sequence. After the baseline condition, the guided competition imagery preceded the self-generated imagery to provide a standardized cognitive framework before participants generated their own sport-specific mental representations. This sequence was adopted to reduce inter-condition variability and facilitate consistent engagement with the imagery task across participants. During all motor imagery conditions, participants remained seated and stationary with their eyes closed, and were instructed to refrain from any overt motor execution. No concurrent EMG or accelerometry was recorded; therefore, subtle muscle activity or micromovements cannot be completely excluded. To minimize potential movement-related contamination, EEG recordings were subjected to the standardized preprocessing and artifact-control procedures described below, including ICA-based correction and semi-automatic artifact inspection using predefined criteria.

Continuous EEG recordings were obtained during all four experimental conditions (Figure 1).

**Figure 1:**
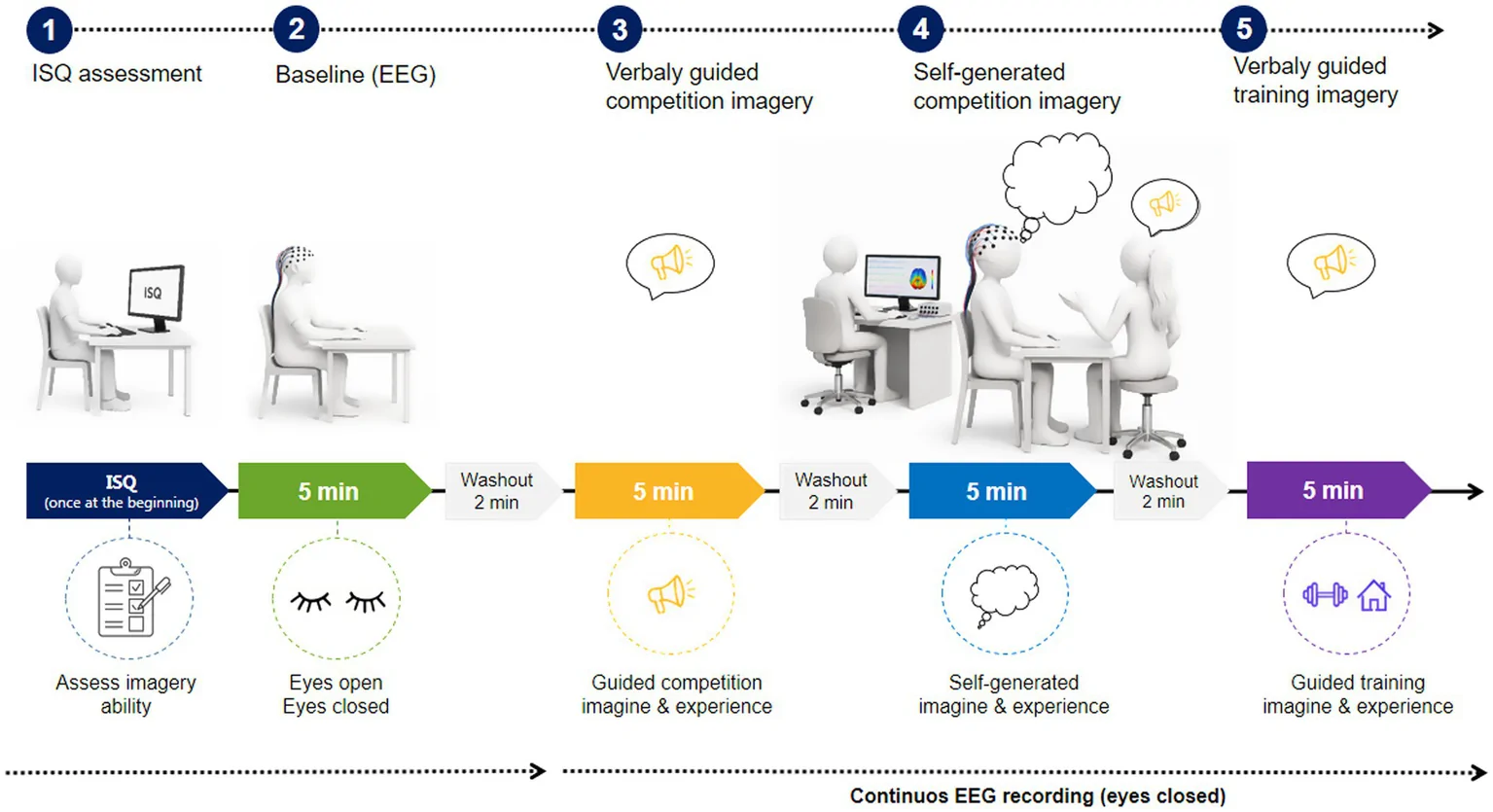
Experimental procedure. Prepared by the authors, with technical and programming assistance provided by OpenAI’s ChatGPT (GPT-4o).

### 2.3 EEG recording

Continuous electroencephalographic (EEG) activity was recorded using 16 active scalp electrodes connected to a 24-bit V-Amp/FirstAmp amplifier (Brain Products GmbH, Gilching, Germany). Electrode placement followed the international 10–10 system and included Fp1, Fp2, F5, F3, Fz, F4, F6, FC5, FC1, FC2, FC6, C3, C1, Cz, C2, and C4. Recordings were obtained using a 128-channel BrainCap configured according to the international 10–10 electrode placement system (Seeck et al., 2017), although only the electrodes of interest were analyzed. EEG signals were sampled at 512 Hz. Electrode impedances were maintained below 10 kΩ at all electrode sites. The electrode positioned at Fpz, located at the prefrontal midline, was used to derive the average reference. During EEG acquisition, an online 60-Hz notch filter and an 8–20 Hz band-pass filter were applied solely for real-time signal visualization and monitoring of signal quality. These online visualization settings did not define the frequency range of the EEG data used for subsequent spectral analyses. The continuous EEG data were retained for offline preprocessing and spectral analysis.

Offline preprocessing was performed using BrainVision Analyzer software (Version 2.3.0; Brain Products GmbH, Germany). The continuous EEG signals were first filtered using a zero-phase band-pass filter from 0.5 to 50 Hz, followed by a 60-Hz notch filter to attenuate power-line interference. The resulting broadband signal was then used for artifact correction and subsequent spectral analysis. Thus, the frequency range used for the spectral analyses was determined by the offline 0.5–50 Hz filter rather than by the 8–20 Hz online visualization filter (see Figure 2).

**Figure 2:**
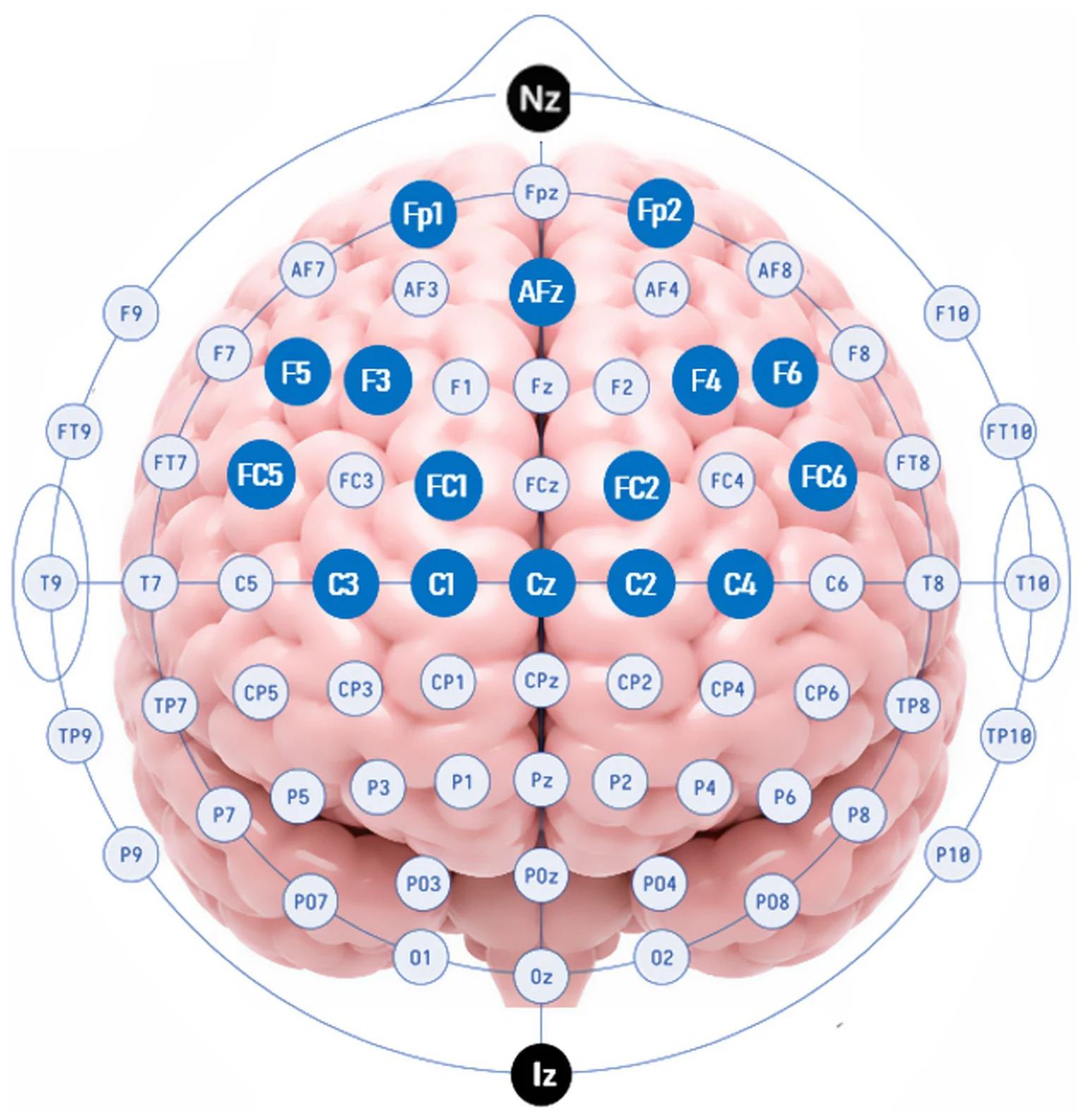
Electrode placement based on the international 10–10 EEG system. Prepared by the authors, with technical and programming assistance provided by OpenAI’s ChatGPT (GPT-4o).

Ocular artifacts were corrected using semi-automatic Independent Component Analysis (ICA). For both participants, ICA was performed on the entire continuous EEG recording using all 16 recorded channels and the Infomax Restricted Biased algorithm. Blinks were detected using the slope-based detection algorithm, with Fp1 used as the vertical ocular-activity channel and Fp2 as the horizontal ocular-activity channel. Components relevant to ocular activity were identified based on the squared correlation with the respective ocular-activity channel, using a 30% threshold for the percentage of total squared correlation. A maximum of 60 blink events was considered by the detection procedure. ICA was performed using 512 steps with a convergence bound of 1 × 10–7.

For the surfer, 16 independent components were computed from 280.56 s of continuous EEG data, and the decomposition converged after 94 iterations following 7 restarts. Components 0 and 1 were identified as predominantly ocular and removed by setting them to zero. For the skateboarder, 16 independent components were computed from 341.75 s of continuous EEG data, and the decomposition converged after 146 iterations following 66 restarts. Components 12, 13, and 14 were identified as predominantly ocular and removed. Thus, the same ICA procedure and detection criteria were applied to both participants, whereas component removal was determined according to the characteristics of each individual EEG recording.

Following ICA correction, semi-automatic artifact inspection was performed using the Raw Data Inspector in BrainVision Analyzer. The same predefined criteria were applied across all experimental conditions. These included a maximum allowed voltage step of 50 μV/ms for the gradient criterion, a maximum difference of 150 μV within a 200-ms interval for the difference criterion, and an amplitude range of −150 to +150 μV. Artifact detection was performed using a temporal window extending from 200 ms before to 800 ms after each detected artifact event. No amplitude, gradient, low-activity, or user-defined artifacts were identified in any of the recreational surfer imagery recordings, and no artifacts were detected in the difference criterion during the imagery recordings. A small difference-related artifact was identified during the recreational surfer baseline recording (0.025% of the recording). For the professional skateboarder, amplitude-related artifacts accounted for 0.810% of the verbally guided training imagery recording, 0.010% of the verbally guided competition imagery recording, and 6.250% of the self-generated competition imagery recording; no other artifact types were detected. All artifact-marked segments were excluded from subsequent spectral analyses. Detailed quality-control information is provided in Supplementary Table S1. After artifact correction and rejection, the continuous EEG recordings were segmented into consecutive 2-s epochs for spectral analysis. Absolute spectral power was estimated using Welch’s method for the theta (>4–≤8 Hz), alpha (>8–≤13 Hz), and beta (>13–≤30 Hz) frequency bands. Only data remaining after the artifact-control procedures were included in the spectral analyses.

#### 2.3.1 EEG data analysis

##### 2.3.1.1 Frontal alpha asymmetry (FAA)

FAA was calculated as the difference between right- and left-frontal alpha power (FAA = F4 − F3). Given the inverse relationship between alpha power and cortical activation, negative FAA values reflect relatively greater right-frontal cortical activation, whereas positive values reflect relatively greater left-frontal cortical activation.

##### 2.3.1.2 Frontal beta asymmetry (FBA)

To evaluate high-frequency cortical arousal and active cognitive engagement, Frontal Beta Asymmetry (FBA) was calculated within the beta band FBA utilized the same homologous electrode pair (F3 and F4) and was calculated as: FB = F4 − F3. Unlike alpha activity, beta-band power is directly and positively correlated with local cortical activation (higher beta power reflects increased neural firing and active processing). Consequently, the interpretation of FBA is inverse to that of FAA.

##### 2.3.1.3 Frontal midline theta (fmθ)

Frontal midline theta (fmθ) was analyzed to monitor cognitive load, working memory engagement, and top-down attentional allocation during the imagery protocols. Theta-band spectral power was extracted from the midline frontal electrode (Fz). This electrode was recorded within the multi-channel montage relative to a common reference, providing spatial and reference control across the scalp. Fmθ was therefore assumed as a scalp-level electrophysiological marker associated with cognitive-control processes. Higher fmθ values reflect increased cognitive demand and conscious attentional monitoring, whereas lower fmθ values reflect lower executive strain and neural efficiency.

##### 2.3.1.4 Event-related spectral perturbation (ERSP) and sensorimotor markers

To validate the occurrence of kinesthetic motor imagery and assess sensorimotor cortex activation, event-related spectral perturbation (ERSP) was calculated for both beta (13–30 Hz) and theta (4–8 Hz) frequency bands across all experimental conditions relative to the resting baseline (C3, Cz, and C4 channels). Spectral power changes were computed as percentage variation: ERSP (%) = ^P^condition – ^P^baseline/^P^baseline × 100. Where ^P^condition represents absolute power during the specific imagery modality and ^P^baseline represents baseline power. Negative values denote Event-Related Desynchronization (ERD), reflecting localized cortical excitability and active motor simulation, whereas positive values denote event-related synchronization (ERS), reflecting cortical inhibition or elevated cognitive load.

## 3 Results

### 3.1 Sport imagery questionnaire (SIQ)

The recreational (amateur) surfer obtained a global SIQ score of 147 points (mean = 4.90), indicating an overall moderate-to-high use of sport imagery. The highest scores were observed in Cognitive Specific imagery (mean = 5.17; raw score = 31) and Cognitive General imagery (mean = 5.00; raw score = 30), both classified as frequent imagery use. The remaining dimensions, Motivational Specific, Motivational General-Arousal, and Motivational General-Mastery, showed moderate imagery use (means ranging from 4.67 to 4.83), suggesting that imagery was employed primarily for technical rehearsal and tactical preparation rather than for motivational regulation. In contrast, the six-time World Cup Skateboarder Champion demonstrated substantially greater imagery engagement across nearly all dimensions, achieving a global SIQ score of 173 points (mean = 5.77). The athlete reported frequent imagery use in four of the five subscales, including Cognitive General (mean = 6.00), Motivational Specific (mean = 6.00), Motivational General-Arousal (mean = 5.00), and particularly Motivational General-Mastery, which reached the maximum possible score (mean = 7.00; raw score = 42). Although Cognitive Specific imagery (mean = 4.83) was slightly lower, it remained within the moderate range, indicating consistent use of imagery for skill rehearsal. Overall, comparison of the two athletes revealed distinct imagery profiles. Whereas the recreational surfer relied predominantly on cognitive imagery related to technical execution, the world champion exhibited a broader and more intensive imagery repertoire, integrating cognitive rehearsal with strong motivational functions, particularly those associated with confidence, emotional control, resilience, and mastery.

### 3.2 Frontal alpha asymmetry (FAA)

Frontal alpha asymmetry (FAA) values for both athletes are presented in Table 1. The elite skateboarder exhibited negative FAA values across all imagery conditions, indicating a consistent pattern of right frontal dominance. FAA decreased in absolute negative magnitude from −2.814 at baseline to −0.007 during verbally guided training imagery, −0.275 during verbally guided competition imagery, and −0.005 during self-generated competition imagery. The recreational surfer showed a different pattern. Baseline FAA was slightly positive (+0.227); during verbally guided training imagery, FAA remained close to baseline (+0.290), but, in contrast, verbally guided competition imagery produced a more pronounced positive FAA (+1.010). During self-generated competition imagery, however, FAA became negative (−0.290).

**Table 1:** Frontal alpha asymmetry (FAA) (μV^2^/Hz).

	Professional skateboarder	Recreational surfer
Baseline	−2.814	0.227
Verbally guided training imagery	−0.007	0.290
Verbally guided competition imagery	−0.275	1.010
Self-generated competition imagery	−0.005	−0.290

### 3.3 Frontal beta asymmetry (FBA)

Frontal beta asymmetry (FBA) was calculated using the same frontal electrode pair (F4 – F3). Unlike alpha activity, beta power is positively associated with cortical activation; therefore, positive FBA values indicate relatively greater right frontal activation, whereas negative values indicate greater left frontal activation. FBA values for both athletes across the experimental conditions are presented in Table 2. At baseline, the elite skateboarder exhibited leftward beta asymmetry (FBA = −7.850) and the recreational surfer a rightward pattern (FBA = +0.942). During imagery, the skateboarder’s FBA values approached zero across all conditions (−0.008, −1.114, and −0.051 for verbally guided training, verbally guided competition, and self-generated competition imagery, respectively). In contrast, the recreational surfer exhibited FBA shifting slightly leftward during training imagery (−1.024) and rightward during both guided competition (+6.170) and self-generated competition imagery (+5.004).

**Table 2:** Frontal beta asymmetry (FBA) (μV^2^/Hz).

	Professional skateboarder	Recreational surfer
Baseline	−7,85,049	0,941,983
Verbally guided training imagery	−0.008	−1.024
Verbally guided competition imagery	−1.114	6.170
Self-generated competition imagery	−0.051	5.004

### 3.4 Frontal midline theta (fmθ)

Frontal midline theta (fmθ), recorded at electrode Fz, was analyzed to examine changes in executive control and attentional engagement during the imagery tasks. Fmθ values for both athletes across the experimental conditions are presented in Table 3. The elite skateboarder exhibited stable fmθ values across the imagery conditions, with only a small increase during verbally guided training imagery (from 0.052 to 0.348 μV^2^/Hz), followed by values close to baseline during guided and self-generated competition imagery (0.090 and 0.016 μV^2^/Hz, respectively). The fmθ values of the recreational surfer showed a decrease from baseline during verbally guided training imagery (0.789 to 0.093 μV^2^/Hz), increasing during verbally guided competition imagery (to 2.565 μV^2^/Hz), and returning toward baseline during self-generated competition imagery (0.489 μV^2^/Hz).

**Table 3:** Frontal midline theta (fmθ) (μV^2^/Hz).

	Professional skateboarder	Recreational surfer
Baseline	0.052	0.789
Verbally guided training imagery	0.348	0.093
Verbally guided competition imagery	0.090	2.565
Self-generated competition imagery	0.016	0.489

### 3.5 Event-related spectral perturbation (ERSP) and sensorimotor validation

Relative power changes (ERSP) in Beta (13–30 Hz) and Theta (4–8 Hz) bands were evaluated to validate the presence of kinesthetic motor imagery (Table 4). The recreational surfer demonstrated sensorimotor Beta ERD exclusively during verbally guided training imagery (−22.6%), accompanied by a + 53.8% Theta ERS. In contrast, competition imagery modalities produced marked Beta ERS (+111.6% in guided and +42.1% in self-generated imagery) along with elevated Theta power (+84.9%) in the guided modality. The elite skateboarder displayed a distinct sensorimotor pattern, characterized by robust Beta ERD during competition imagery (−30.1% in verbally guided and −61.4% in self-generated imagery). Concurrently, Theta power exhibited pronounced desynchronization during both competition conditions (−61.7% and −86.4%, respectively). Conversely, verbally guided training imagery induced Beta ERS (+41.0%) and Theta ERS (+35.2%).

**Table 4:** Spectral power (μV^2^/Hz) and event-related spectral perturbation (ERSP, %) for beta and theta bands across experimental imagery conditions.

Condition	Beta power (μV^2^/Hz)	Beta ERSP (%)	Theta power (μV^2^/Hz)	Theta ERSP (%)
Recreational surfer				
Baseline	2.666	—	2.248	—
Verbally guided training imagery	2.063	−22.6%	3.457	+53.8%
Verbally guided competition imagery	5.642	+111.6%	4.156	+84.9%
Self-generated competition imagery	3.789	+42.1%	1.293	−42.5%
Professional skateboarder				
Baseline	0.886	—	0.283	—
Verbally guided training imagery	1.249	+41.0%	0.382	+35.2%
Verbally guided competition imagery	0.619	−30.1%	0.108	−61.7%
Self-generated competition imagery	0.342	−61.4%	0.038	−86.4%

ERSP (%) represents the relative percentage change in spectral power relative to baseline. Negative ERSP values indicate Event-Related Desynchronization (ERD/cortical activation), whereas positive values indicate event-related synchronization (ERS/cortical inhibition).

## 4 Discussion

### 4.1 Frontal alpha asymmetry (FAA)

The inclusion of the baseline condition provides an important reference for interpreting the task-related changes in frontal alpha asymmetry. The two athletes exhibited distinct patterns of FAA across motor imagery conditions, suggesting that the neural configuration observed during imagery was not simply a stable characteristic of athletic expertise, but was modulated by the specific imagery context.

The elite skateboarder exhibited negative FAA values across all imagery conditions, indicating relatively greater right-frontal activation according to the adopted FAA convention. However, the magnitude of the asymmetry was substantially reduced relative to baseline. FAA decreased in absolute negative magnitude from −2.814 at baseline to −0.007 during verbally guided training imagery, −0.275 during verbally guided competition imagery, and −0.005 during self-generated competition imagery. Thus, although the direction of the asymmetry remained rightward during imagery, the pronounced rightward asymmetry observed at baseline was replaced by a substantially more balanced frontal configuration during mental simulation. This pattern suggests that the imagery conditions were associated with a redistribution of relative frontal alpha activity rather than a simple maintenance of the baseline lateralization pattern.

The recreational surfer showed a different pattern. Baseline FAA was slightly positive (+0.227), indicating a modest relative left-frontal activation. During verbally guided training imagery, FAA remained close to baseline (+0.290), suggesting little task-related change in frontal asymmetry. In contrast, verbally guided competition imagery produced a more pronounced positive FAA (+1.010), representing a shift toward greater relative left-frontal activation. During self-generated competition imagery, however, FAA became negative (−0.290), indicating a reversal toward relatively greater right-frontal activation. Thus, unlike the skateboarder, whose FAA moved toward symmetry across all imagery conditions, the recreational surfer exhibited condition-dependent modulation of frontal asymmetry, particularly during competition imagery.

The contrast between the two athletes is therefore better characterized as a difference in the task-related modulation of frontal lateralization rather than simply as a difference in the direction of FAA. The elite skateboarder showed a pronounced baseline asymmetry that became substantially attenuated during imagery, whereas the recreational surfer displayed a relatively symmetric baseline configuration followed by condition-specific shifts in lateralization. The elite skateboarder’s convergence toward near-zero FAA across imagery conditions may be particularly relevant to theories of neural efficiency as well as models of transient hypofrontality and the flow experience (Dietrich, 2004, 2006) associated with optimal performance states. Nevertheless, baseline-referenced changes in FAA were measured in the present study, theoretically reflecting lateralized frontal dynamics, attentional/cognitive-control processes, and task-related modulation. Although the design of the present study does not allow direct inferences regarding these theoretical concepts, it is reasonable to speculate on an association with such a background.

These findings raise the possibility that motor expertise and familiarity with imagery may influence how frontal cortical resources are dynamically configured during mental simulation. In highly trained athletes who routinely incorporate mental imagery into their training, motor representations may become sufficiently automated to reduce the need for continuous executive supervision. Without the constraints imposed by an externally guided script, the athlete appeared to adopt a more self-organized and integrated imagery strategy, resembling the experiential “letting it happen” mode described by Swann et al. (2016). Such a pattern may reflect a shift toward internally generated sensorimotor simulation supported by efficient cortical processing rather than by increased executive engagement.

The greater leftward FAA observed in the recreational surfer during verbally guided competition imagery may be compatible with increased involvement of cognitive control, attentional regulation, and the construction and monitoring of internally generated task representations. Rather than accessing automated motor representations, the athlete appeared to consciously monitor and regulate the imagined performance, consistent with the “making it happen” mode proposed by Swann et al. (2016). This interpretation is supported by the substantial leftward asymmetry observed under externally guided conditions, which may reflect greater reliance on top-down cognitive control during imagery construction. However, given the single-subject nature of the study, this interpretation should be considered exploratory rather than evidence of a specific underlying cognitive mechanism.

Conversely, the reversal toward negative FAA during self-generated competition imagery suggests that removal of continuous verbal guidance may have altered the balance of frontal processing, potentially facilitating greater reliance on internally generated visuospatial or sensorimotor representations. One possible explanation is that the absence of continuous verbal guidance reduced analytical monitoring and facilitated greater immersion in visuospatial and episodic representations (Budnik-Przybylska et al., 2021) of previous surfing experiences. Although the underlying neural mechanisms cannot be established from the present case study, this shift raises the possibility that less structured imagery protocols may promote greater access to sensorimotor representations even in individuals with lower levels of competitive expertise or limited experience with systematic mental imagery training. Consequently, imagery structure itself, in addition to athletic expertise, might represent an important determinant of the neural processes recruited during mental simulation (Moran et al., 2012).

Overall, the FAA findings suggest that athletic expertise may be associated not simply with a particular direction of frontal asymmetry, but with a distinct capacity to modulate frontal lateralization according to the demands and structure of the imagery task. The contrast between guided and self-generated competition imagery further suggests that imagery structure itself may influence the balance of frontal processes recruited during motor simulation.

### 4.2 Frontal beta asymmetry (FBA)

Frontal beta asymmetry revealed a distinct pattern of task-related modulation between the two athletes. At baseline, the elite skateboarder exhibited pronounced leftward beta asymmetry (FBA = −7.850), whereas the recreational surfer showed a near-symmetric but slightly rightward pattern (FBA = +0.942). During imagery, however, the skateboarder’s FBA values approached zero across all conditions (−0.008, −1.114, and −0.051 for verbally guided training, verbally guided competition, and self-generated competition imagery, respectively), indicating a substantial attenuation of the marked baseline asymmetry. In contrast, the recreational surfer exhibited condition-dependent changes, with FBA shifting slightly leftward during training imagery (−1.024) and becoming strongly rightward during both guided competition (+6.170) and self-generated competition imagery (+5.004).

The baseline-referenced pattern is particularly informative. In the elite skateboarder, all imagery conditions were associated with large positive changes in FBA relative to baseline, reflecting a marked reduction in the pronounced leftward beta asymmetry observed at rest. Importantly, these values should be interpreted as changes in relative hemispheric beta activity rather than as direct evidence of a reduction in absolute beta power. The near-zero FBA values during imagery indicate greater hemispheric balance, but the underlying absolute beta activity must be considered separately when interpreting changes in cortical engagement.

The recreational surfer, in contrast, demonstrated a pronounced rightward beta asymmetry specifically during competition imagery. The magnitude of this effect was substantially greater than that observed during baseline and training imagery. This pattern may be compatible with increased recruitment of frontal processes associated with attentional allocation, cognitive monitoring, and maintenance of internally generated task representations during competitive motor imagery. The persistence of rightward beta asymmetry during self-generated competition imagery suggests that the removal of continuous verbal guidance did not eliminate the task-related frontal asymmetry. However, the present single-subject design does not permit a definitive attribution of this pattern to executive control or cognitive workload. These findings complement the FAA results and suggest that motor expertise influences not only hemispheric specialization during motor imagery but also the magnitude of executive resources recruited to sustain mental simulation. Whereas the recreational athlete exhibited greater frontal beta activity while imagining competitive situations, the world-class skateboarder maintained comparatively low frontal beta power across all imagery conditions. This pattern is consistent with assumptions that highly skilled performers accomplish complex cognitive and motor tasks through a more economical allocation of cortical resources, relying less on effortful executive control and more on well-established sensorimotor representations (Cheron et al., 2016; Babiloni et al., 2010; Del Percio et al., 2008; Del Percio et al., 2009b; Engel and Fries, 2010).

Indeed, beta oscillations have consistently been associated with sustained attention, executive control, and the maintenance of internally generated task representations (Engel and Fries, 2010; Spitzer and Haegens, 2017). From this perspective, mentally simulating an entire competitive event likely imposed greater executive and attentional demands than imagining isolated sport-specific actions. Notably, right frontal dominance persisted during self-generated competition imagery, suggesting that the removal of continuous verbal guidance did not substantially reduce cognitive workload. Rather, the participant appeared to continue relying on top-down executive mechanisms to construct, maintain, and monitor an internally generated representation of the competitive scenario, a process that is consistent with the established role of the prefrontal cortex in goal-directed cognitive control (Guillot et al., 2012; Miller and Cohen, 2001).

The elite skateboarder demonstrated a distinctly different neurophysiological profile. Frontal beta activity decreased substantially across all imagery conditions, reaching its lowest levels during self-generated competition imagery, where both hemispheres displayed minimal absolute beta power. Rather than reflecting reduced task engagement, this attenuation may indicate that internally simulating well-established motor skills required relatively little executive control. Extensive competitive experience, together with systematic mental imagery practice, may have enabled the athlete to access consolidated motor representations with minimal reliance on frontal cognitive resources.

Although the underlying mechanisms cannot be established from the present case study, these findings suggest that externally guided imagery may influence performers differently depending on the degree of motor expertise, skill automatization, and familiarity with systematic mental imagery training. For highly trained athletes, externally guided scripts might primarily facilitate retrieval of already consolidated motor representations, whereas individuals with less competitive experience or limited imagery practice might depend more heavily on executive processes to construct and regulate the imagined performance.

Taken together, the FAA and FBA findings suggest that the two athletes differed not only in the direction of frontal asymmetry but also in how hemispheric balance was modulated from baseline during motor imagery. The elite skateboarder showed attenuation of pronounced baseline asymmetries, resulting in relatively balanced frontal alpha and beta activity during imagery. The recreational surfer, by contrast, showed stronger task-dependent lateralization, particularly during competition imagery. This pattern is compatible with the possibility that extensive motor expertise and imagery familiarity are associated with more economical or less lateralized frontal processing during motor simulation, whereas less experienced performers may recruit more condition-dependent frontal resources. Nevertheless, these interpretations should be regarded as hypothesis-generating given the single-subject nature of the study

### 4.3 Frontal midline theta (fmθ)

Frontal midline theta (fmθ) has consistently been associated with cognitive processes involving sustained attention, working memory, conflict monitoring, and performance monitoring (Cavanagh and Frank, 2014; Mitchell et al., 2008). Indeed, in the present study, Theta-band spectral power was extracted from the midline frontal electrode (Fz), following established theoretical and methodological frameworks that validate absolute power at Fz as a core index of midfrontal cognitive control (Cavanagh and Frank, 2014). Rather than representing a nonspecific marker of mental effort, fmθ is increasingly understood as an oscillatory correlate of the dynamic recruitment of attentional and executive processes during goal-directed cognition (Cavanagh and Frank, 2014; Mitchell et al., 2008). Within the present case study, the fmθ findings provide complementary information to the alpha and beta asymmetry measures, revealing distinct patterns of frontal oscillatory modulation across athletes and imagery conditions.

Marked differences were observed between the two athletes when fmθ was considered relative to baseline. The elite skateboarder exhibited comparatively low and stable fmθ values across the imagery conditions, with only a modest increase during verbally guided training imagery (from 0.052 to 0.348 μV^2^/Hz), followed by values close to baseline during guided and self-generated competition imagery (0.090 and 0.016 μV^2^/Hz, respectively). In contrast, the recreational surfer exhibited substantial condition-dependent modulation, with fmθ decreasing from baseline during verbally guided training imagery (0.789 to 0.093 μV^2^/Hz), increasing markedly during verbally guided competition imagery (to 2.565 μV^2^/Hz), and returning toward baseline during self-generated competition imagery (0.489 μV^2^/Hz).

The pronounced fmθ response observed in the recreational surfer during verbally guided competition imagery represents the largest task-related deviation from baseline among the conditions examined. Given the established association between frontal midline theta and attentional control, working memory, and performance monitoring (Cavanagh and Frank, 2014; Hsieh and Ranganath, 2014), this pattern is compatible with greater recruitment of frontal cognitive processes while constructing and maintaining a complex competitive scenario under continuous verbal guidance. Importantly, however, the present data cannot establish that the increase in fmθ directly represents greater cognitive effort or executive workload. Rather, it indicates a greater modulation of frontal theta activity under this particular imagery condition.

Interestingly, fmθ decreased substantially during self-generated competition imagery in the recreational surfer, despite the absence of continuous verbal guidance. This reduction may indicate a different organization of the imagery process, in which internally generated representations were maintained with less pronounced frontal midline theta modulation. Such a pattern is compatible with the possibility that self-generated imagery reduced the requirement for externally driven attentional regulation, although this interpretation remains speculative given the single-subject design. Importantly, the reduction in fmθ should not be interpreted as evidence of reduced task engagement, as successful motor imagery can be supported by distributed sensorimotor processes without requiring a large increase in frontal midline theta activity (Guillot et al., 2012; Hardwick et al., 2018; Budnik-Przybylska et al., 2021).

The elite skateboarder showed a contrasting pattern, characterized by relatively stable fmθ across the imagery conditions and minimal deviation from baseline during competition imagery. This profile suggests that the different imagery conditions did not produce substantial changes in frontal midline theta activity in this athlete. Considering the athlete’s extensive competitive experience and reported use of imagery, one possible interpretation is that highly consolidated motor representations may have reduced the need for substantial task-related modulation of frontal oscillatory activity during the simulation of familiar competitive actions. However, this interpretation should be considered hypothesis-generating rather than demonstrative, as the present design does not permit direct inference regarding cognitive workload, neural efficiency, or the degree of executive control engaged during imagery.

An additional finding was the slightly higher fmθ observed during verbally guided training imagery in the elite skateboarder (0.348 μV^2^/Hz) compared with both guided competition imagery (0.090 μV^2^/Hz) and self-generated competition imagery (0.016 μV^2^/Hz). Although the magnitude of this difference was small in absolute terms, the pattern may indicate that the neural demands of imagery depend not only on expertise but also on the nature and familiarity of the representation being generated. Training-related imagery may involve greater contextual reconstruction, attentional orientation, or integration of specific situational elements, whereas competition imagery in a highly experienced athlete may more readily activate consolidated representations developed through repeated practice and competitive exposure (Guillot et al., 2012; Ericsson and Harwell, 2019).

The fmθ findings therefore complement the FAA and FBA results by demonstrating that the two athletes differed not simply in the absolute magnitude of frontal oscillatory activity, but in the extent and direction of task-related modulation relative to baseline. The recreational surfer showed pronounced modulation during verbally guided competition imagery, whereas the elite skateboarder maintained a comparatively stable frontal theta profile across conditions. Taken together, these findings are compatible with the hypothesis that expertise and imagery modality may influence how strongly frontal attentional and executive networks are modulated during motor imagery. Nevertheless, the present results should be interpreted as descriptive, within-individual neurophysiological patterns rather than evidence of a direct relationship between expertise and reduced cognitive effort or enhanced neural efficiency.

### 4.4 Neurophysiological markers of motor imagery

The occurrence of kinesthetic motor imagery may be observed due to sensoriomotor markers used in the present study, while illustrating marked differences in neural dynamics between the professional skateboarder and the recreational surfer. Standard sensorimotor markers dictates that effective motor imagery manifests as Beta desynchronization (ERD) over sensorimotor cortices, reflecting increased neuronal excitability (Pfurtscheller and Lopes da Silva, 1999; Triana-Guzman et al., 2022). The professional skateboarder demonstrated a neurophysiological signature that might be associated with his high-performance level. During self-generated competition imagery a prominent Beta ERD (−61.4%) coupled with pronounced Theta ERD (−86.4%) was observed. While Beta ERD validates sensorimotor activation during mental rehearsal (Muraoka et al., 2024), the concomitant Theta suppression aligns with the *Neural Efficiency Hypothesis* (Babiloni et al., 2010). Theta ERS is widely associated with frontal working memory demands and external attentional control; thus, its marked desynchronization in the elite athlete suggests an automated, low-effort cognitive state akin to “flow” during self-directed rehearsal. Conversely, the recreational surfer required external verbal guidance to recruit sensorimotor circuits (Beta ERD: −22.6%), a process that has been associated with executive attention as evidenced by high Theta ERS (+53.8%). During competitive imagery, the recreational surfer displayed Beta ERS, that might indicate the motor cortex inhibition or cognitive overload. Overall, these multi-band ERSP patterns suggest that while the professional athlete efficiently recruits motor representations during autonomous imagery practice, the recreational surfer relies on external guidance and experiences performance-inhibiting cognitive load under simulated competitive contexts.

## 5 Final considerations

This case study provides preliminary neurophysiological evidence that motor imagery is influenced by both the mode of imagery generation and the athlete’s level of expertise. By simultaneously examining frontal alpha asymmetry (FAA), frontal beta asymmetry (FBA), and frontal midline theta (fmθ), the present findings suggest that these oscillatory measures provide complementary information about the neural dynamics associated with motor imagery. FAA and FBA characterize relative hemispheric differences in frontal alpha- and beta-band activity, respectively, whereas fmθ provides an additional index of frontal oscillatory engagement commonly associated with attentional control, conflict monitoring, and other executive processes. Rather than representing isolated neural markers, these measures may capture partially overlapping components of the cognitive processes involved in mental simulation.

The inclusion of the baseline condition further indicates that the two athletes differed not only in the absolute magnitude of their oscillatory measures but also in how frontal activity was modulated during imagery relative to their resting state. The elite skateboarder exhibited substantial attenuation of baseline frontal asymmetries during imagery, together with relatively stable and low fmθ across the imagery conditions. In contrast, the recreational surfer showed more pronounced condition-dependent modulation, particularly during verbally guided competition imagery, which was characterized by increased FAA, FBA, and fmθ relative to baseline. This pattern suggests that the neural response to motor imagery may depend on the interaction between imagery structure, task content, and individual expertise.

Behavioral findings further contribute to this interpretation. Although the world champion skateboarder reported greater overall imagery use than the recreational surfer, his strongest imagery function was Motivational General–Mastery rather than Cognitive Specific imagery. This profile may suggest that, with increasing expertise, imagery becomes increasingly integrated with self-regulation, competitive preparation, and mastery-related processes rather than being restricted to detailed rehearsal of specific movements. As motor skills become highly consolidated through extensive practice, imagery may consequently serve functions beyond motor acquisition, including the maintenance of confidence, attentional stability, emotional regulation, and competitive readiness.

Importantly, the SIQ findings converged with the electrophysiological observations. The elite athlete reported greater imagery use while exhibiting relatively attenuated frontal beta asymmetry and lower frontal midline theta during self-generated imagery. This pattern is compatible with the possibility that highly consolidated motor representations can be accessed with comparatively limited frontal oscillatory modulation. In contrast, the recreational athlete exhibited stronger condition-dependent changes in frontal beta and theta activity, particularly during competition imagery, suggesting that the generation of complex competitive scenarios may involve greater modulation of frontal cognitive processes. Nevertheless, these interpretations remain exploratory and cannot establish differences in cognitive workload or neural efficiency from the present case-study design.

Finally, the present findings suggest that the neural organization of motor imagery may depend not only on expertise but also on the instructional modality and the nature of the imagined content. Guided competition imagery was associated with the most pronounced frontal oscillatory modulation in the recreational surfer, whereas self-generated competition imagery produced a different pattern characterized by reduced fmθ and a reversal of FAA relative to guided competition imagery. In the elite skateboarder, by contrast, imagery was associated with attenuation of baseline frontal asymmetries and relatively stable fmθ.

Together, these observations highlight the potential value of combining behavioral measures of imagery use with electrophysiological markers to characterize individual differences in the neural dynamics of motor imagery across different levels of athletic expertise. Given the single-subject design, these findings should be considered hypothesis-generating and warrant confirmation in larger samples using segment-level statistical analyses and appropriately controlled experimental designs. Moreover, while FAA, FBA, and fmθ delineate the dynamics during motor imagery, the accompanying ERSP analysis across beta and theta bands provides the necessary sensorimotor validation that kinesthetic motor imagery occurred. Together, these multi-band spectral metrics suggest the imagetic of the professional skateboarder may be characterized by reduced frontal executive burden alongside localized sensorimotor engagement (ERD), whereas recreational surfer dynamics relies on higher cognitive-attentional control. This multi-band approach reinforces that the neural organization of motor imagery is a complex interplay between athlete expertise, task structure, and underlying sensorimotor activation.

### 5.1 Limitations and perspectives

It should be highlighted that the present findings are specific to these two participants and caution should be exercised when extrapolating results to broader athlete populations, as well as against treating individual-specific phenomena as general neural mechanisms. Moreover, as pointed out throughout the text, limitations extend beyond sample size and must be emphasized. These include the cross-sport comparison, the inability to isolate expertise from individual characteristics, the fixed condition order, variations in prior imagery experience, the absence of repeated sessions, the reliance on frontal scalp measures, the lack of behavioral or phenomenological measures collected after each imagery condition, and the exploratory nature of the proposed EEG markers.

Although the present investigation is limited by its case-study design and the aforementioned aspects, it introduces a theoretically grounded framework that may guide future research examining the neurophysiology of motor imagery across different expertise levels, sports, and training interventions. Future studies involving larger samples should determine whether the combined assessment of FAA, FBA, and fmθ can serve as an integrated set of neurophysiological biomarkers for motor expertise, imagery quality, and cognitive efficiency. Such an approach may ultimately contribute to individualized mental training protocols and to the objective monitoring of neurocognitive adaptations during athletic preparation, rehabilitation, and return-to-performance programs.

More broadly, this framework supports the emerging perspective that the neurophysiology of motor imagery should not be interpreted through isolated oscillatory markers, but rather through the coordinated interaction of complementary neural systems governing information processing, executive control, and attentional regulation. Understanding these interactions may represent an important step toward establishing objective neurophysiological signatures of expertise and optimizing evidence-based applications of mental imagery in sport.

## Data Availability

The raw data supporting the conclusions of this article will be made available by the authors, without undue reservation.

## References

[ref1] Babiloni C. Marzano N. Infarinato F. Iacoboni M. Rizza G. Aschieri P. . (2010). Neural efficiency of experts' brain during judgment of actions: a high-resolution EEG study in elite and amateur karate athletes. Behav. Brain Res. 207, 466–475. doi: 10.1016/j.bbr.2009.10.034, 19891991

[ref2] Budnik-Przybylska D. Kastrau A. Jasik P. Kaźmierczak M. Doliński Ł. Syty P. . (2021). Neural oscillation during mental imagery in sport: an Olympic sailor case study. Front. Hum. Neurosci. 15:669422. doi: 10.3389/fnhum.2021.669422, 34140884 PMC8205149

[ref3] Cavanagh J. F. Frank M. J. (2014). Frontal theta as a mechanism for cognitive control. Trends Cogn. Sci. 18, 414–421. doi: 10.1016/j.tics.2014.04.012, 24835663 PMC4112145

[ref4] Cheron G. Petit G. Cheron J. Leroy A. Cebolla A. Cevallos C. . (2016). Brain oscillations in sport: toward EEG biomarkers of performance. Front. Psychol. 7:246. doi: 10.3389/fpsyg.2016.00246, 26955362 PMC4768321

[ref5] Cisek P. Kalaska J. F. (2010). Neural mechanisms for interacting with a world full of action choices. Annu. Rev. Neurosci. 33, 269–298. doi: 10.1146/annurev.neuro.051508.135409, 20345247

[ref6] Cumming J. Williams S. E. (2012). “The role of imagery in performance,” in The Oxford Handbook of Sport and Performance Psychology, ed. Murphy S. M. (Hoboken, NJ: John Wiley & Sons), 213–232.

[ref7] Del Percio C. Babiloni C. Bertollo M. . (2009a). Visuo-attentional and sensorimotor alpha rhythms are related to visuo-motor performance in athletes. Hum. Brain Mapp. 30, 3527–3540. doi: 10.1002/hbm.20776, 19350556 PMC6870716

[ref8] Del Percio C. Babiloni C. Marzano N. . (2009b). Neural efficiency of athletes' brain for upright standing: a high-resolution EEG study. Brain Res. Bull. 79, 193–200. doi: 10.1016/j.brainresbull.2009.02.001, 19429191

[ref9] Del Percio C. Iacoboni M. Lizio R. . (2011). Functional coupling of parietal α rhythms is enhanced in athletes before visuomotor performance: a coherence electroencephalographic study. Neuroscience 175, 198–211. doi: 10.1016/j.neuroscience.2010.11.031, 21144884

[ref10] Del Percio C. Rossini P. M. Marzano N. . (2008). Is there a "neural efficiency" in athletes? A high-resolution EEG study. Neuroimage 42, 1544–1553. doi: 10.1016/j.neuroimage.2008.05.061, 18602484

[ref11] Dietrich A. (2004). Neurocognitive mechanisms underlying the experience of flow. Conscious. Cogn. 13, 746–761. doi: 10.1016/j.concog.2004.07.002, 15522630

[ref12] Dietrich A. (2006). Transient hypofrontality as a mechanism for the psychological effects of exercise. Psychiatry Res. 145, 79–83. doi: 10.1016/j.psychres.2005.07.033, 17081621

[ref13] Engel A. K. Fries P. (2010). Beta-band oscillations—signalling the status quo? Curr. Opin. Neurobiol. 20, 156–165. doi: 10.1016/j.conb.2010.02.015, 20359884

[ref14] Ericsson K. A. Harwell K. W. (2019). Deliberate practice and proposed limits on the effects of practice on the acquisition of expert performance: why the original definition matters and recommendations for future research. Front. Psychol. 10:2396. doi: 10.3389/fpsyg.2019.02396, 31708836 PMC6824411

[ref15] Fomin R. N. Collins C. C. (2022). “Neuroscience approach to performance,” in NSCA'S Essentials of Sport Science, eds. French D. N. Torres-Ronda L. (Champaign, IL: Human Kinetics). IL: chap 27

[ref16] Guillot A. Di Rienzo F. MacIntyre T. Moran A. Collet C. (2012). Imagining is not doing but involves specific motor commands: a review of experimental data related to motor inhibition. Front. Hum. Neurosci. 6:247. doi: 10.3389/fnhum.2012.00247, 22973214 PMC3433680

[ref17] Hardwick R. M. Caspers S. Eickhoff S. B. Swinnen S. P. (2018). Neural correlates of action: comparing meta-analyses of imagery, observation, and execution. Neurosci. Biobehav. Rev. 94, 31–44. doi: 10.1016/j.neubiorev.2018.08.003, 30098990

[ref18] Hatfield B. D. Kerick S. E. (2007). “The psychology of superior sport performance: a cognitive and affective neuroscience perspective,” in Handbook of Sport Psychology, eds. Tenenbaum G. Eklund R. C.. 3rd ed (Hoboken, NJ: John Wiley & Sons), 84–109.

[ref19] Hsieh L. T. Ranganath C. (2014). Frontal midline theta oscillations during working memory maintenance and episodic encoding and retrieval. NeuroImage 85, 721–729. doi: 10.1016/j.neuroimage.2013.08.003, 23933041 PMC3859771

[ref20] Jeannerod M. (1994). The representing brain: neural correlates of motor intention and imagery. Behav. Brain Sci. 17, 187–202. doi: 10.1017/S0140525X00034026

[ref21] Jeannerod M. (2001). Neural simulation of action: a unifying mechanism for motor cognition. NeuroImage 14, S103–S109. doi: 10.1006/nimg.2001.0832 11373140

[ref22] Kays J. L. Hurley R. A. Taber K. H. (2012). The dynamic brain: neuroplasticity and mental health. J. Neuropsychiatry Clin. Neurosci. 24, 118–124. doi: 10.1176/appi.neuropsych.24.2.118 22772660

[ref23] Miller E. K. Cohen J. D. (2001). An integrative theory of prefrontal cortex function. Annu. Rev. Neurosci. 24, 167–202. doi: 10.1146/annurev.neuro.24.1.167, 11283309

[ref24] Mitchell D. J. McNaughton N. Flanagan D. Kirk I. J. (2008). Frontal-midline theta from the perspective of hippocampal theta. Prog. Neurobiol. 86, 156–185. doi: 10.1016/j.pneurobio.2008.09.005, 18824212

[ref25] Moran A. Guillot A. MacIntyre T. Collet C. (2012). Re-imagining motor imagery: building bridges between cognitive neuroscience and sport psychology. Br. J. Psychol. 103, 224–247. doi: 10.1111/j.2044-8295.2011.02068.x, 22506748

[ref26] Morris T. Spittle M. Watt A. P. (2005). Imagery in sport. Hum. Kinetics.

[ref27] Munroe-Chandler K. J. Guerrero M. D. (2017). Psychological Imagery in Sport and Performance. New York, NY: Oxford University Press.

[ref28] Muraoka Y. Iwama S. Ushiba J. (2024). Neurofeedback-induced desynchronization of sensorimotor rhythm elicits pre-movement downregulation of intracortical inhibition that shortens simple reaction time in humans: a double-blind, sham-controlled randomized study. Imaging Neurosci (Camb). 2:imag-2-00383. doi: 10.1162/imag_a_00383, 40800510 PMC12315730

[ref29] Nakata H. Yoshie M. Miura A. Kudo K. (2010). Characteristics of the athletes' brain: evidence from neurophysiology and neuroimaging. Brain Res. Rev. 62, 197–211. doi: 10.1016/j.brainresrev.2009.11.006, 19944119

[ref30] Pfurtscheller G. Lopes da Silva F. H. (1999). Event-related EEG/MEG synchronization and desynchronization: basic principles. Clin. Neurophysiol. 110, 1842–1857. doi: 10.1016/s1388-2457(99)00141-8, 10576479

[ref31] Seeck M. Koessler L. Bast T. Leijten F. Michel C. Baumgartner C. . (2017). The standardized EEG electrode array of the IFCN. Clin. Neurophysiol. 128, 2070–2077. doi: 10.1016/j.clinph.2017.06.254, 28778476

[ref32] Spitzer B. Haegens S. (2017). Beyond the status quo: a role for Beta oscillations in endogenous content (re)activation. eNeuro 4:ENEURO.0170-17.2017. doi: 10.1523/ENEURO.0170-17.2017, 28785729 PMC5539431

[ref33] Swann C. Keegan R. Crust L. Piggott D. (2016). Psychological states underlying excellent performance in professional golfers: "letting it happen" versus "making it happen.". Psychol. Sport Exerc. 23, 101–113. doi: 10.1016/j.psychsport.2015.10.008

[ref34] Triana-Guzman N. Orjuela-Cañon A. D. Jutinico A. L. Mendoza-Montoya O. Antelis J. M. (2022). Decoding EEG rhythms offline and online during motor imagery for standing and sitting based on a brain-computer interface. Front. Neuroinform. 16:961089. doi: 10.3389/fninf.2022.961089, 36120085 PMC9481272

[ref35] White A. Hardy L. (1998). An in-depth analysis of the uses of imagery by high-level slalom canoeists and artistic gymnasts. Sport Psychol. 12, 387–403. doi: 10.1123/tsp.12.4.387

[ref36] Wolpert D. M. Miall R. C. Kawato M. (1998). Internal models in the cerebellum. Trends Cogn. Sci. 2, 338–347. doi: 10.1016/S1364-6613(98)01221-2, 21227230

[ref37] Yang J. F. Scholz J. P. (2005). Learning a throwing task is associated with differential changes in the use of motor abundance. Exp. Brain Res. 163, 137–158. doi: 10.1007/s00221-004-2149-x, 15657698

[ref38] Yarrow K. Brown P. Krakauer J. W. (2009). Inside the brain of an elite athlete: the neural processes that support high achievement in sport. Nat. Rev. Neurosci. 10, 585–596. doi: 10.1038/nrn2672, 19571792

