# Supplementary material for: Neural oscillatory activity during guided and self-generated motor imagery: a comparative case study of a six-time World Cup skateboarder champion and an experienced recreational surfer
Source: Front Hum Neurosci. 2026 Oct 7;20:1964730. doi: 10.3389/fnhum.2026.1964730 (PMC13683666; doi:10.3389/fnhum.2026.1964730)
Supplement: Supplementary file 1 [file Table_1.DOCX]

**Supplementary Table S1. Semi-Automatic EEG Artifact Inspection Using BrainVision Analyzer**

| **Participant** | **Condition** | **Recording duration (s)** | **Low activity (%)** | **Amplitude (%)** | **Difference (%)** | **Gradient (%)** | **User-defined (%)** | **Total artifact-marked (%)** |
| --- | --- | --- | --- | --- | --- | --- | --- | --- |
| Recreational Surfer | Baseline | 458.0 | 0.000 | 0.000 | 0.025 | 0.000 | 0.000 | **0.025** |
| Recreational Surfer | Verbally guided training imagery | 713.125 | 0.000 | 0.000 | 0.000 | 0.000 | 0.000 | **0.000** |
| Recreational Surfer | Verbally guided competition imagery | 490.500 | 0.000 | 0.000 | 0.000 | 0.000 | 0.000 | **0.000** |
| Recreational Surfer | Self-generated competition imagery | 427.000 | 0.000 | 0.000 | 0.000 | 0.000 | 0.000 | **0.000** |
| Professional Skateboarder | Baseline | 341.750 | 0.000 | 0.000 | 0.000 | 0.000 | 0.000 | **0.000** |
| Professional Skateboarder | Verbally guided training imagery | 477.438 | 0.000 | 0.810 | 0.000 | 0.000 | 0.000 | **0.810** |
| Professional Skateboarder | Verbally guided competition imagery | 612.000 | 0.000 | 0.010 | 0.000 | 0.000 | 0.000 | **0.010** |
| Professional Skateboarder | Self-generated competition imagery | 383.375 | 0.000 | 6.250 | 0.000 | 0.000 | 0.000 | **6.250** |

*Note:* **Artifact-inspection parameters.** Semi-automatic inspection was performed using the Raw Data Inspector in BrainVision Analyzer. Sixteen EEG channels were inspected (C3, F5, F6, FC1, FC2, FC5, FC6, Fp1, Fp2, C1, C2, C4, Cz, F3, F4, and Fz). The predefined criteria were: maximum voltage step of 50 μV/ms for gradient detection; maximum difference of 150 μV within a 200-ms interval for difference detection; and amplitude limits of −150 to +150 μV. For each detected artifact, a temporal window extending from 200 ms before to 800 ms after the event was marked as bad. The same criteria were applied across all recordings and experimental conditions.

**Note.** Percentages represent the proportion of each recording marked by the corresponding artifact criterion in BrainVision Analyzer. Artifact-marked segments were excluded from subsequent spectral analyses.
